# Isolation and Identification of a High-Yield Ethyl Caproate-Producing Yeast From *Daqu* and Optimization of Its Fermentation

**DOI:** 10.3389/fmicb.2021.663744

**Published:** 2021-05-31

**Authors:** Guangsen Fan, Pengxiao Liu, Xu Chang, Huan Yin, Liujie Cheng, Chao Teng, Yi Gong, Xiuting Li

**Affiliations:** ^1^Beijing Advanced Innovation Center for Food Nutrition and Human Health, Beijing Technology and Business University (BTBU), Beijing, China; ^2^School of Food and Health, Beijing Technology and Business University (BTBU), Beijing, China; ^3^Institute of Brewing and Bioenergy, Angel Yeast Co., Ltd., Hubei, China

**Keywords:** *Baijiu*, ethyl caproate, identification, fermentation optimization, *Clavispora lusitaniae*

## Abstract

*Baijiu* is an important fermented product in China. A yeast named YX3307 that is capable of producing a large amount of ethyl caproate (EC) was isolated from *Daqu*, a crude fermentation starter for *Baijiu*. This yeast was identified as *Clavispora lusitaniae* on the basis of its morphological properties, physiological and biochemical characteristics, and 26S rDNA sequence. Single-factor experiments were conducted to obtain the optimum fermentation conditions for EC production by YX3307. The highest EC yield (62.0 mg/L) from YX3307 was obtained with the following culture conditions: inoculum size 7.5% (v/v), seed cell age 30 h, sorghum hydrolysate medium (SHM) with a sugar content of 10 Brix and an initial pH of 6.0; incubation at 28°C with shaking at 180 rpm for 32 h; addition of 10% (v/v) anhydrous ethanol and 0.04% (v/v) caproic acid at 32 and 40 h, respectively, static culture at 20°C until 72 h. YX3307 synthesized more EC than ethyl acetate, ethyl lactate, ethyl butyrate, and ethyl octanoate. An intracellular enzyme or cell membrane enzyme was responsible for EC synthesis. YX3307 can produce many flavor compounds that are important for high-quality *Baijiu*. Thus, it has potential applications in improving the flavor and quality of *Baijiu*.

## Introduction

*Baijiu*, a typical Chinese traditional fermented food, is made from sorghum, wheat, and/or rice by a complex fermentation process using natural mixed-culture starters (named *Daqu*) (Fan et al., 2020a). One of those starters is *Daqu*, which harbors many microorganisms for *Baijiu* brewing, and is used as a saccharifying and fermenting agent for *Baijiu* production. *Daqu* is made from cereals such as wheat, barley, and peas by spontaneous solid-state fermentation in an open environment (Fan et al., 2020a). During *Daqu* production, the raw materials are colonized by networks of microorganisms from the environment. Thus, *Daqu* used is a natural microbial library of bacteria, fungi, and yeasts that play important roles during the *Baijiu* fermentation (Hu et al., 2020). Because *Daqu* is produced using a range of different preparation methods and in different environments, the microbial composition of *Daqu* produced in different places can be quite different (Zuo et al., 2020). Therefore, it is important to screen and identify microorganisms from different kinds of *Daqu*, especially the functional microorganisms that could improve *Baijiu* quality (Fan et al., 2018b).

Strong-flavor *Baijiu* accounts for more than 70% of total *Baijiu* consumed in China because of its fragrant flavor, soft mouthfeel, and long aftertaste (Liu and Sun, 2018). Although the unique taste of strong-flavor *Baijiu* is the result of the interaction of many flavor substances, ethyl caproate (EC) with its apple-like flavor is the characteristic flavor substance. This compound determines the quality and aroma profile of strong-flavor *Baijiu* (Chen et al., 2014, 2016; Tan et al., 2016; Song et al., 2020). There is a clear requirement for a certain content of EC (0.40–2.80 g/L for different grades of strong-flavor *Baijiu*) in the standard of strong-flavor *Baijiu* (China Light Industry Press, and People’s Republic of China Professional Standard, 2006). As the main flavor substance, EC is the main factor that restricts the yield of excellent-grade strong-flavor *Baijiu* (Fan and Qian, 2006).

Like other esters, EC in *Baijiu* is mainly derived from microbial metabolism (Fan et al., 2020b). There are two ways that EC can be produced: synthesis from caproic acid and ethanol catalyzed by lipase or esterase; and catalysis of ethanol and caproyl-coenzyme A by ethanol hexanoyl transferase (Liu et al., 2004; Chen et al., 2014, 2016; Takahashi et al., 2017). Previous studies have shown that EC is mainly produced during the later stage of *Baijiu* brewing (Chen et al., 2014). Therefore, to increase the content of EC in strong-flavor raw *Baijiu* (without blending), the period of *Baijiu* brewing is often extended. This practice not only leads to high grain consumption and low production efficiency, but also results in the production of unpleasant flavor compounds (Chen et al., 2014, 2016). A similar problem has been encountered in sake, but it was solved by isolating a strain of sake yeast with a high yield of EC (Ichikawa et al., 1991; Arikawa et al., 2000; Aritomi et al., 2004; Takahashi et al., 2017). This provides a reference to solve the problem of the low EC content in strong-flavor *Baijiu*. That is, there is the potential to improve quality and strengthen the EC content by using functional microorganism(s) with high yields of EC in the fermentation process. In the future, strong-flavor *Baijiu* may be produced by combining and regulating the growth of a limited number of functional microorganisms, just like sake.

Among diverse microorganisms such as yeasts, molds, and bacteria, some yeasts are known to harbor the above two EC synthesis pathways (Chen et al., 2016). Therefore, screening for a yeast strain with a high yield of EC is of great significance to increase the EC content in strong-flavor *Baijiu*, as has been achieved for sake. Although some researchers have attempted to isolate yeasts with high yields of EC from the *Baijiu* brewing environment, there has been limited success in isolating a suitable strain (Wang et al., 2013). In view of these considerations, we focused on *Daqu* to isolate and characterize a yeast capable of producing a large amount of EC. In this study, a yeast with high-yields of EC was isolated and purified from *Daqu* using a traditional screening method. The yeast was identified and the fermentation conditions were optimized for EC production.

## Materials and Methods

### Materials and Reagents

*Daqu* samples were collected from different *Baijiu* brewing enterprises in China (detailed information is provided in Supplementary Table 1). The EC (HPLC grade) was obtained from Roche. The EC and tetraoctanol standards were purchased from Sigma (St. Louis, MO, United States). All other chemicals were of analytical grade and commercially available unless otherwise stated.

### Media Preparation

Yeast extract peptone dextrose (YPD) medium, Wallerstein laboratory nutrient agar (WL) medium, and sorghum hydrolysate medium (SHM) were prepared as described in our previous report (Fan et al., 2018b). The media for physiological and biochemical tests were prepared according to standard methods (Kurtzman et al., 2011).

### Screening for Yeast With a High-Yield of EC

A mixture of 25 g *Daqu* sample and 225 mL sterilized ddH_2_O was diluted into three gradients, from 10^–4^ to 10^–6^. After serial dilution, 100-μL aliquots from each gradient suspension were spread onto YPD plates. The plates were incubated at 30°C and monitored daily for 5 days until yeast colonies developed. Single colonies showing different morphologies were picked and purified from the plates and microscopic examination was performed after lawn growth. Then, each isolated colony was inoculated into a YPD medium slant tube and stored at −80°C. Each yeast strain (1 × 10^6^ cells/mL) was inoculated into 30 mL SHM and cultured at 28°C with shaking at 180 r/min for 24 h. After adding precursors [4% (v/v) ethanol and 0.02% (v/v) caproic acid], the cultures were incubated for a further 36 h. The cultures were centrifuged at 13,000 × *g* for 10 min, the supernatant was filtered, and then the EC content was determined by gas chromatography–mass spectrometry (GC-MS). Each yeast strain in the experiments was analyzed in parallel three times. The yeast producing the largest amount of EC was selected for further study.

### Identification of Yeast

The screened strain was identified on the basis of its colony morphology, cell morphology, physiological and biochemical characteristics, and 26S rDNA sequence, as described by Kurtzman et al. (2011) and Fan et al. (2018b). Phenotypic characterization was also carried out with Biolog GEN III MicroPlates (Biolog) according to the manufacturer’s instructions. Each selected strain was first grown on nutrient yeast glycerol agar medium (NYGA, 5 g/L peptone, 3 g/L yeast extract, 20 g/L glycerol, and 15 g/L agar) for 48 h at 28°C, then on solid Biolog Dehydrated Growth agar for 24 h at 28°C. Using a cotton-tipped swab, fresh colonies were transferred into vials containing Inoculating Fluid A. After the density of the inoculum was adjusted to a transmittance of 95–98% (as measured using a turbidimeter), 100 μL prepared inoculum was dispensed into each well of the Biolog MicroPlate. The plate was incubated at 28°C and assayed using a MicroStation 2 Reader (Biolog) at various times.

### Tolerance Features of YX3307

In experiments testing the temperature and pH range for growth, and tolerance to glucose, NaCl, ethanol, caproic acid, and EC, the cell density of YX3307 was monitored by measuring optical density at 560 nm (OD_560_), as described in a previous report (Fan et al., 2018b). The levels of each factor in the growth and tolerance tests are shown in Supplementary Table 2.

### Optimization of Culture Conditions

Various culture conditions (Supplementary Table 3), including pH, shaking speed, temperature, ethanol content, caproic acid content, inoculum age, sugar content, time of ethanol addition, inoculum size, time of caproic acid addition, and culture time, were optimized for EC production under submerged fermentation in SHM. For these analyses, we used the classical approach of altering only one variable per test, whereby a single factor was adjusted while others were kept constant. After optimization, the optimal conditions were used in subsequent fermentations. Before the yeast was inoculated into the fermentation medium, the yeast cells were activated in 30 mL YPD at 28°C for 24 h. After preculture, 0.1 mL yeast suspension (OD_560_ is about 45–50) was used to inoculate 30 mL SHM as described above. Thereafter, the EC content was determined by GC-MS.

### Preliminary Study on EC Synthesis

To explore the primary mechanism of EC synthesis by the selected yeast, four groups of experiments were designed. In group A, SHM was adjusted to pH 4.3 (the lowest pH in the fermentation experiments), and ethanol and caproic acid were added at optimal times and concentrations as determined in the optimization experiments. In group B, fermentation was carried out according to the optimal conditions. In group C, fermentation was carried out according to the optimal conditions. In addition, the yeast was removed by centrifugation before adding the earlier precursor, and then fermentation proceeded according to the optimal conditions. In group D, opposite to group C, before adding the earliest precursor, the yeast was obtained and washed with saline solution, then transferred to fresh SHM for fermentation under optimal conditions. In group D, the EC content in the first fermentation broth before adding the precursor was also determined.

### Characteristics of Ester Production

To systematically study the ester production characteristics of the screened yeast, ethanol and different acids (acetic acid, lactic acid, butyric acid, caproic acid, and octanoic acid) were added to the SHM as precursors. Each acid was added with ethanol separately. The selected acids were precursors of important ester compounds in *Baijiu*. The fermentation method was the same as that used to screen for yeasts with a high yield of EC. The esters in different samples were detected by GC-MS.

### Aroma Production

To explore the functional characteristics of yeasts, the aroma-producing characteristics of the selected yeasts were analyzed as reported elsewhere (Fan et al., 2019). After preculture as described above, yeast cells (initial density, 1 × 10^6^ cells/mL) were inoculated into 30 mL SHM and cultured at 28°C with shaking at 180 r/min for 72 h. Then, the sample was centrifuged at 13,000 × *g* for 10 min at 4°C. The aroma compounds in the supernatant were analyzed by headspace solid-phase microextraction-GC-MS (HS-SPME-GC-MS). Uninoculated SHM served as the control.

### Analytical Methods

The OD of cells was detected at 560 nm with an ultraviolet spectrophotometer (TU-1901; Purkinje General Instrument Co., Ltd., Pinggu, Beijing, China). After pretreatment, the EC content in the fermentation medium was determined by GC-MS as reported previously (Fan et al., 2018b). Briefly, 2 mL of each sample was centrifuged, then mixed with an equal volume of heptane and incubated for 5 min. The organic phase was transferred to a centrifuge tube containing anhydrous sodium sulphite, and was kept at −20°C overnight to remove water from the sample. After filtering through a 0.45-μm nylon filter, the sample was analyzed by GC-MS. The GC-MS conditions were as follows: initial temperature, 50°C for 2 min, increased to 180°C for 2 min, then to 230°C for 2 min. The vaporization chamber temperature and the inlet temperature were 250°C, the carrier gas was high-purity helium, and a DB-WAX capillary column was used. The injection mode was split (split ratio of 37:1) and the injection volume was 1 μL. An electron impact ionization ion source with a 70 eV of electron energy was used, the scanning range was 30–550 amu, and the ion source temperature was 230°C (Fan et al., 2018b). The EC content was calculated by comparison with a calibration curve obtained by serial dilution of an EC standard solution. The aroma compounds were analyzed by HS-SPME-GC-MS according to Fan et al. (2018a) with minor changes. The aroma compounds in the fermentation mixture were extracted by 50/30-μm divinylbenzene/carboxen on polydimethylsiloxane (DVB/CAR on PDMS)-coated fibers. The 15-mL headspace vial contained (Supelco, Inc., Bellefonte, PA, United States) 5 mL supernatant, 2 g NaCl, and 10 μL of 0.5 g/L tetraoctanol solution. The vial was covered with a cap and kept in a water bath at 50°C for 10 min. Then, a SPME needle was inserted to extract aroma compounds for 30 min at 50°C. The extraction head was inserted into the GC-MS inlet for desorption at 250°C for 5 min. The GC-MS conditions were as follows: initial temperature, 40°C for 3 min, increased to 100°C for 5 min, then to 150°C for 3 min, and then to 280°C for 6 min. These analyses were conducted in the splitless mode. The mass spectra of aroma compounds with positive and negative matches higher than 800 with those from National Institute of Standards and Technology library (NIST) are reported. For each identified compound, the amount was calculated as the ratio of the mass concentration of tetraoctanol to the mass concentration of the compound.

### Statistical Analysis

Statistical differences among treatment groups were detected by one-way ANOVA (*P* < 0.05) followed by Tukey test. Assays were conducted at least in triplicate, and the reported values correspond to the mean value and its standard deviation. Data were processed and analyzed using SPSS 24.0 (IBM Corp., New York, NY, United States), OriginPro 9.1 (Origin-Lab, Northampton, MA, United States), and Excel 2016 (Microsoft, United States).

## Results and Discussion

### Screening for Yeast Capable of Producing EC

A total of 147 yeasts were isolated from strong-flavor *Daqu*. Each yeast was inoculated into SHM to test its ability to produce EC. Although most yeasts were able to synthesize EC, the yield was relatively low, consistent with other reports (Wang et al., 2013). Previous studies have shown that most yeasts isolated from the *Baijiu*-producing environment can produce EC, but most of them have a low EC production capacity (Wu et al., 2013). This may explain the low EC content in strong-flavor raw *Baijiu*, and why it is necessary to prolong the fermentation period to increase the EC content. Only 19 yeasts produced EC at concentrations exceeding 0.5 g/L (Supplementary Table 4). Among those yeasts, strain YX3307 was chosen as the target strain for further experiments because it produced significantly more EC than did the other strains.

### Identification of Yeast YX3307

**FIGURE 1:**
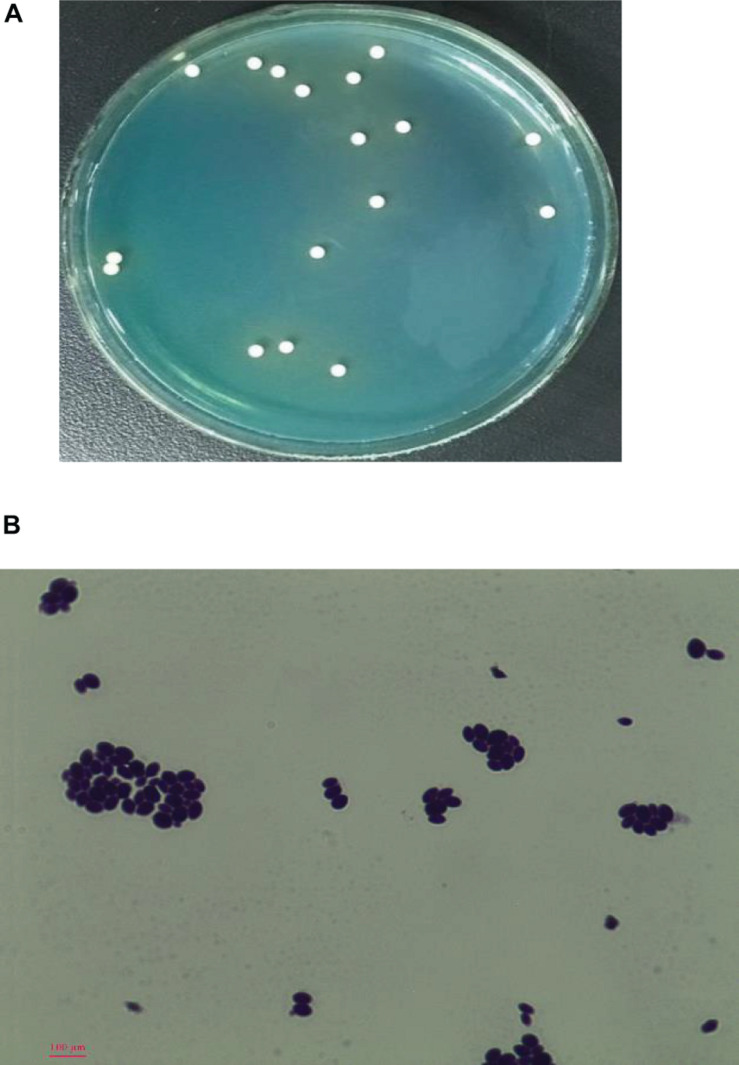
Colony morphology on WL **(A)** and cell morphology **(B)** of strain YX3307 captured by microscopy (10 × 40). Cell was stained by the crystal violet method. The asexual budding reproduction occurred at the ends of the cells was highlighted in the blue circle.

After growth for 72 h at 28°C on WL agar, the colonies of strain YX3307 were milky white, flat with a little protuberance in the center, wet and smooth with an entire margin, and diameter of 2–3 mm (Figure 1A). Meanwhile, the WL agar changed from blue to light yellow where this strain grew (Figure 1A). The cells of YX3307 were fusiform to ovoid, occurring as a single cell or parental bud pairs. Asexual budding reproduction was observed at the ends of the cells as shown in Figure 1B.

As shown in Table 1, when YX3307 was fermented in the presence of glucose, D-galactose, maltose, saccharose, cellobiose, or raffinose, aerogenesis, and acid production occurred. In the presence of inulin or trehalose, only acid was produced by YX3307. The yeast YX3307 was unable to ferment lactose, melibiose, D-xylose, soluble starch, or arabinose, as indicated by the lack of acid or gas production. YX3307 readily utilized glucose, galactose, L-sorbose, cellobiose, maltose, saccharose, trehalose, melezitose, raffinose, D-ribose, L-rhamnose, D-sorbitol, glycerol, D-mannitol, ethyl alcohol, ribitol, DL-lactic acid, or D-glucosamine. It grew more slowly when lactose, inulin, L-arabinose, D-xylose, citric acid, succinic acid, D-glucuronic acid, or myricetrin was supplied as the sole carbon source. YX3307 was unable to assimilate melibiose, soluble starch, D-arabinose, erythritol, galactitol, inositol, methyl alcohol, α-methyl-glucoside, or hexadecane.

**TABLE 1:** Physiological and biochemical characteristics tests.

**Sugar fermentation test**					
Sugars	Characteristics of YX3307	Sugars	Characteristics of YX3307	Sugars	Characteristics of YX3307
D-glucose	Acid and gas production	D-galactose	Acid and gas production	D-maltose	Acid and gas production
D-saccharose	Acid and gas production	D-lactose	No acid, no gas	Inulin	Acid production, no gas
Melibiose	No acid, no gas	Cellobiose	Acid and gas production	D-xylose	No acid, no gas
D-raffinose	Acid and gas production	D-trehalose	Acid production, no gas	Soluble starch	No acid, no gas
D-arabinose	No acid, no gas				
**Carbon source assimilation test**					
Carbon sources	Characteristics of YX3307	Carbon sources	Characteristics of YX3307	Carbon sources	Characteristics of YX3307
D-glucose	+++	D-galactose	+++	L-sorbose	++
Cellobiose	++	D-lactose	+	D-maltose	+++
Melibiose	−	D-saccharose	++	D-trehalose	+++
Melezitose	++	D-raffinose	++	Inulin	+
Soluble starch	−	D-arabinose	−	L-arabinose	+
D-ribose	++	L-rhamnose	++	D-xylose	+
Erythritol	−	Galactitol	−	D-sorbitol	+++
Glycerol	++	Inositol	−	D-mannitol	+++
Ethyl alcohol	++	Ribitol	++	Methyl alcohol	−
Citric acid	+	DL-lactic acid	++	Succinic acid	+
D-glucuronic acid	+	α-methyl-glucoside	−	Myricetrin	+
D-glucosamine	++	Hexadecane	−		
**Nitrogen source assimilation test**					
Nitrogen sources	Characteristics of YX3307	Nitrogen sources	Characteristics of YX3307	Nitrogen sources	Characteristics of YX3307
Potassium nitrate	+	Cadaverine dihydrochloride	+++	Ethylamine hydrochloride	++
Sodium nitrite	−	L-phenylalanine	+	L-lysine	+++
Urea	++	Ammonium sulfate	+	Ammonium nitrite	−
**Vitamin requirement test**					
Riboflavin	++	Folic acid	++	Pyridoxine hydrochloride	++
Myo-inositol	++	Calcium pantothenate	++	Thiamine	+++
Niacin	+++	*p*-aminobenzoic acid	++	Biotin	++
**Other tests**					
Starch hydrolysis test	−	Methyl red test	−	Indole test	+
Voges-Proskauer test	−	Hydrogen sulfide test	−	Urea test	+
TTC medium test	−	Citrate test	−	Litmus milk test	−
Gelatin liquidized test	−	Lipase activity test	−	CGB agar test	−
Formation of extracellular amyloid compounds test	−	Diazonium blue B color reaction	−	Tolerance of 1% of acetic acid test	−
DOPA medium test	+	Splitting of arbutin test	+	Actinomycin tolerance test	0–25 μg/mL
Sodium chloride osmolarity test	0–15% (w/v)	Glucose osmolarity test	0–80% (w/v)	Caproic acid tolerance test	0.06% (v/v)
Ethanol tolerance test	0–6% (v/v)	Growth temperature range	15–50°C (optimum 25°C)	Growth pH range	1.0–11.0 (optimum pH 6.0)
Ethyl caproate tolerance test	0–1200 mg/L				

*“+,” positive response, the more the plus sign indicated the more positive it is; “−,” negative response.*

When cadaverine dihydrochloride, ethylamine hydrochloride, L-lysine, or urea was used as the sole nitrogen source, YX3307 grew very well. It was also able to grow when potassium nitrate, L-phenylalanine, or ammonium sulfate was supplied as the sole nitrogen source. However, it could not grow in the presence of sodium nitrite or ammonium nitrite. YX3307 also grew well when supplied with test vitamins, especially thiamine (VB_1_) and niacin (VB_3_).

The starch hydrolysis test, methyl red test, Voges-Proskauer test, hydrogen sulfide test, tetrazolium indicator medium (TTC medium) test, citrate test, litmus milk test, gelatin liquification test, lipase activity test, canavanine-glycine-bromothymol blue (CGB) agar test, formation of extracellular amyloid compounds test, diazonium blue B color reaction and tolerance to 1% acetic acid test were negative. The indole test, urea test, melanin synthesis on L-dihydroxyphenylalanine (DOPA) medium test, and splitting of arbutin test were positive. The physiological and biochemical characteristics of YX3307 resembled those of *C. lusitaniae*, consistent with the Biolog results (Supplementary Figure 1). These analyses of the physiological and biochemical characteristics of YX3307 provided information about its metabolic spectrum, and consequently, its potential functions.

After amplification and sequencing a 26S rDNA fragment from YX3307, the sequence was submitted to NCBI database^1^ under the accession number MW440554, and searches were conducted to find the closest homolog. The fragment showed 100% sequence similarity with the partial 26S rDNA sequence from *C. lusitaniae* ACE6 (EF063129.1). As shown in the phylogenetic tree, YX3307, *C. lusitaniae* MB141 (KF830171.1) and *C. lusitaniae* GSWW10 (EF536910.1) clustered into a branch, indicating that they were very closely related (Supplementary Figure 2).

According to its morphological, physiological, and biochemical characteristics, and based on the results of the Biolog and molecular analyses, YX3307 was identified as *C. lusitaniae*. Previous studies have shown that *C. lusitaniae* occurs in a broad of substrates of plant and animal origin, as well as in wastes and clinical specimens (Zhang et al., 2010). Although *C. lusitaniae* was first reported by Pappagianis et al. (1979), few studies have focused on this yeast (1979). *C. lusitaniae* in strong-flavor *Baijiu* was first reported by Wei et al. (2013), but has subsequently been isolated from other flavors of *Baijiu*. Although some analyses of microbial diversity in *Baijiu* have noted the presence and importance of *C. lusitaniae* (Wen and Li, 2018; Cui X. L. et al., 2019; Du et al., 2019; Zhou et al., 2020), few studies have explored its function in *Baijiu*, especially its role in EC production.

### Tolerance Features of YX3307

The *Baijiu* brewing environment is complex, and the pH, temperature, water activity, and contents of ethanol and other compounds change during the fermentation time. These factors affect the growth and reproduction of microorganisms in this environment. The microorganisms that can survive in this environment have certain adaptive characteristics. Therefore, we analyzed the tolerance of yeast YX3307 to provide a reference for its application in *Baijiu* brewing. As shown in Table 1, YX3307 showed good environmental adaptability because of its tolerance features. YX3307 was able to grow in the presence of a certain amount of actinomycin; the maximum tolerable concentration was 25 μg/mL. This indicated that YX3307 has some ability to adapt to the presence of other microorganisms, especially actinomycetes, in the *Baijiu* brewing environment. YX3307 was also tolerant to high temperature (growth was observed at 50°C) and was able to grow over a wide pH range (pH 1–11), indicating that this strain can adapt to the changing conditions during the *Baijiu* brewing process. YX3307 also showed strong tolerance to osmotic pressure, and was able to grow in media containing 80% (w/v) glucose and 15% (w/v) NaCl. To explore its ability to synthesize EC, the tolerance of the strain to the precursors, ethanol and caproic acid, and the product, EC, was analyzed. YX3307 was able to tolerate EC at 1,200 mg/L, indicating that it had the potential for high EC production. However, its tolerance to ethanol and caproic acid was relatively low [6% (v/v) and 0.06% (v/v), respectively]. Therefore, to improve EC production, YX3307 could be co-fermented with *Saccharomyces cerevisiae* and caproic acid-producing bacteria, as the yield of EC would increase by the rapid and continuous conversion of ethanol and caproic acid produced by these microorganisms.

### Characteristics of Ester Production

Esters are important flavor compounds in *Baijiu*, and 506 of them have been detected in *Baijiu* to date (Liu and Sun, 2018). Among these esters, ethyl acetate, ethyl lactate, ethyl butyrate, and EC are the main four ethyl esters, and ethyl octanoate is the most important (Fan et al., 2018b; Ma et al., 2020). Although these five ester compounds are present at varying proportions in strong-flavor *Baijiu*, the EC content is low, while those of ethyl lactate and ethyl acetate are generally higher than the standard requirements for strong-flavor *Baijiu*. As a result, the proportion of ester compounds in the raw *Baijiu* is inconsistent, so that it does not meet the standard requirements of strong-flavor *Baijiu*. Now, producers of strong flavor *Baijiu* have dual goals to increase the EC content and decrease the ethyl acetate/ethyl lactate contents (Li J. H. et al., 2019; Song et al., 2019). To explore the ester production characteristics of YX3307, it was inoculated into media containing different precursors (five different acids and ethanol). The results showed that YX3307 mainly synthesized EC and produced a small amount of ethyl acetate, while the other three ester compounds were not detected (Supplementary Table 5). Therefore, YX3307 specifically produces EC. Even when YX3307 produced a large amount of EC, there was no increase ethyl lactate synthesis and very little ethyl acetate was produced. This feature is conducive to solving the current problem of producing strong-flavor *Baijiu* with a low EC content and high content of ethyl acetate/ethyl lactate.

### Optimization of Culture Conditions

In strong-flavor *Baijiu*, EC is the important flavor compound. Sorghum is the main raw material of *Baijiu* brewing. Therefore, SHM was used as the initial fermentation medium to optimize the conditions for YX3307 to produce EC in submerged fermentation.

#### Effect of pH on EC Production

The pH can affect the permeability of yeast cell membranes and the activity of metabolic enzymes. Yeasts employ different metabolic reactions to adapt to the environmental pH. The growth of yeast cells and the type and yield of metabolites vary under different pH conditions (Mattey, 1992). The synthesis of fermentation products can be regulated by adjusting the pH value during fermentation. Therefore, the effect of the initial pH of SHM on the production of EC by yeast YX3307 was investigated. As shown in Supplementary Figure 3, the yield of EC increased first and then decreased as the initial pH of SHM increased. The highest yield of EC was at pH 6, consistent with the optimum pH for YX3307 (Table 1). The yield of EC from YX3307 was slightly higher when the initial pH was alkaline than when the initial pH was acid, because alkaline conditions favored the growth of YX3307. In general, the initial pH of SHM that favored yeast growth also favored EC synthesis; that is, there was a positive correlation between yeast cell growth and EC synthesis as mentioned previously (Dufour et al., 2003). In previous studies, EC was mainly synthesized by a chemical reaction between caproic acid and ethanol when the initial pH of the medium was less than 3 (Zhang et al., 2017). Therefore, if the EC produced at pH 2 or 3 was attributed to chemical synthesis, then less EC was yielded from chemical synthesis than from yeast-catalyzed synthesis (comparing yields between pH 2–3 and 4–9). When the initial pH of SHM was 6.0, the pH value of the fermentation broth was 4.3 at the end of fermentation, consistent with previous report that pH 4.0–4.5 is optimum for EC synthesis via esterification in *Daqu* (Zhang et al., 2017). The initial pH of 6.0 favored the growth of YX3307, and its metabolic activity modified the pH of the growth environment so that it was conducive to EC synthesis. Therefore, the best initial pH of SHM for EC synthesis by YX3307 was 6.0. These results indicate that EC is mainly synthesized by the metabolic processes of YX3307.

#### Effect of Shaking Speed on EC Production

Yeasts are facultative anaerobic microorganisms, and their metabolic pathways differ between aerobic and anaerobic conditions (Panozzo et al., 2002). Previous reports have shown that the types and amounts of metabolites are affected by the amount of dissolved oxygen (Barberel and Walker, 2000). The dissolved oxygen level was regulated by changing the shaking speed to explore its effect on the synthesis of EC by YX3307. As shown in Supplementary Figure 4, EC was produced by YX3307 in the static or shaking state, with higher production levels in the static state than in the shaking state, regardless of the amount of shaking. This differs from the synthesis of ethyl acetate by *Wickerhamomyces anomalus* described in our previous study, where the yield of ethyl acetate was larger in the shaking state than in the static state, and larger with a higher shaking rate than with a low shaking rate (Fan et al., 2018b). These different responses to dissolved oxygen levels are because of the different synthesis mechanisms of ethyl acetate and EC in *W. anomalus* and *C. lusitaniae*. The high yield of ethyl acetate from *W. anomalus* under high dissolved oxygen levels is due to the increased amount of acetyl coenzyme A, the precursor that is converted into ethyl acetate by acyltransferase (Fu et al., 2018). However, in *C. lusitaniae*, low dissolved oxygen levels result in the accumulation of long-chain saturated fatty acids and the inhibition of acetyl CoA carboxylase. Subsequently, acyl CoAs are released from fatty acid synthase, leading to the accumulation of medium-chain fatty acyl CoAs, which increases EC synthesis under low dissolved oxygen levels (Dufour et al., 2003). The biomass of YX3307 was lower in the static state than in the shaking state, and it was higher at high shaking speed (Supplementary Figure 5). Considering the difference in biomass, the dissolved oxygen level more strongly affected EC synthesis by YX3307 than did pH (Supplementary Figure 5). Lower dissolved oxygen levels were conducive to EC synthesis, indicating that this yeast is well-suited for producing EC in the anoxic solid-state fermentation environment of *Baijiu.*

#### Effect of Temperature on EC Production

A previous experimental study showed that temperature affects the number of esters profile (Dufour et al., 2003). As shown in Supplementary Figure 6, the yield of EC first increased and then decreased as the temperature increased. The highest yield of EC was 6.8 mg/L at 20°C. Similar to the effect of dissolved oxygen on EC production, the optimal temperature for EC production was not the optimal temperature for growth of YX3307, nor was it the optimal temperature reported for ethyl acetate synthesis in *W. anomalus* (Fan et al., 2018b). The number of acetate esters increased with increasing temperature, but the number of medium-chain fatty acid esters was not affected, consistent with the results of a previous report (Engan and Aubert, 1977). This provided further evidence that the biosynthesis pathways of the two esters in the two yeasts are different. It is likely that the increased level of acetate esters at higher temperatures is related to the increase in higher alcohols synthesis and alcohol acetyltransferase activity with increased yeast growth, while stimulation of yeast growth prevents acyl CoA accumulation and does not result in increased MCFA esters contents (Dufour et al., 2003). Although a lower temperature was not conducive to the growth of YX3307, it was beneficial for EC biosynthesis.

#### Effect of Ethanol Content on EC Production

**FIGURE 2:**
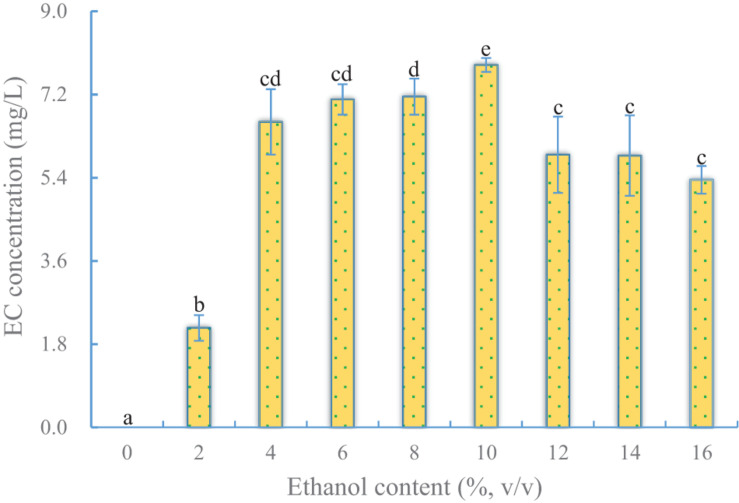
Effect of ethanol content on EC production by YX3307. Same letters in the column indicates that the data do not differ significantly at 5% probability by the Tukey test.

Ethanol is one of the precursors of EC, which is synthesized by an esterifying enzyme. Although YX3307 could produce a little ethanol in SHM, it was insufficient to meet the needs of EC synthesis. Therefore, ethanol was added into SHM to increase EC production. The results showed that the yield of EC first increased and then decreased as the ethanol content in the medium increased (Figure 2). The highest yield of EC was 7.8 mg/L when the ethanol content in the medium was 10%. When less ethanol or no ethanol was added, substantially less EC was produced. This indicated that YX3307 needed ethanol to produce EC. During the *Baijiu* brewing process, YX3307 can produce EC because ethanol is produced by some other microorganisms, such as *S. cerevisiae*. However, a high ethanol content can reduce the synthesis of EC by inhibiting cell growth and enzyme activity. Interestingly, EC synthesis was highest when the ethanol content was slightly higher than the level tolerated by YX3307 (Figure 2 and Table 1). This may be because sufficient biomass of YX3307 accumulated at the early stage before ethanol was added. More importantly, low temperature and dissolved oxygen conditions favored the metabolic pathway of EC synthesis, so that ethanol and caproic acid could be quickly converted into EC before having toxic effects on YX3307 (Table 1). High temperature or aerobic conditions affected YX3307’s tolerance to ethanol (Piper, 1995). YX3307 could tolerate a higher ethanol concentration after optimization of temperature and dissolved oxygen content. That is, the toxicity of ethanol to YX3307 was reduced under low temperature and dissolved oxygen content.

#### Effect of Caproic Acid Content on EC Production

**FIGURE 3:**
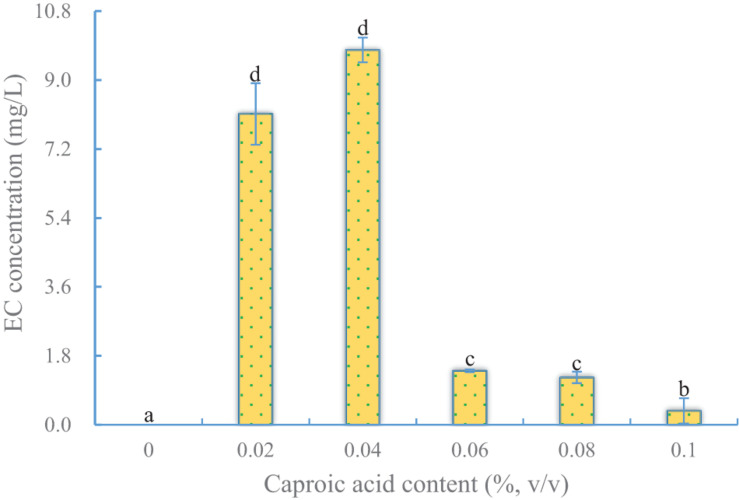
Effect of caproic acid content on EC production by YX3307. Same letters in the column indicates that the data do not differ significantly at 5% probability by the Tukey test.

Because a rate-limiting step in EC synthesis is the abundance of caproic acid, it is an important precursor for EC synthesis by YX3307 (Takahashi et al., 2017). The effect of caproic acid on EC production by YX3307 was similar to that of ethanol, in that the yield of EC increased first and then decreased as the caproic acid content in the medium increased (Figure 3). The optimal caproic acid content for EC production by YX3307 was 0.04% (v/v). This is less than that in another study, and this may reflect differences in caproic acid tolerance between *S. cerevisiae* Y-E and YX3307 (Yuan et al., 2015). In addition, less EC was produced when the caproic acid content was too low or too high because of the lack of precursor or the inhibition of yeast cells, respectively. No EC was produced when no caproic acid was added, different from the case of ethyl acetate production by aroma-producing yeast. This provided further evidence that the pathways of EC synthesis and ethyl acetate synthesis are different in aroma-producing yeast (Fan et al., 2018b).

#### Effect of Inoculum Age on EC Production

Physiological activity differs among cells of different ages, and this affects the types and amounts of metabolites produced. As shown in Supplementary Figure 7, the yield of EC from XY3307 first increased and then decreased as the inoculum age increased. The cells were mainly in the delayed phase before 6 h, during which cells were accumulating enzymes and intermediate metabolites for growth. In addition, the cell density was lower at this stage (Supplementary Figure 8). When cells in this phase were inoculated into SHM, there was a smaller number of cells and their vitality was low. Therefore, after 24 h of culture, there were fewer cells to produce EC. The cells were in the early and middle stages of the logarithmic period during 6–18 h. Although their metabolism was vigorous, the total number of cells was insufficient. Therefore, the yield of EC was still low when cells at these stages were used as the inoculum. During 18–30 h, the cells were in middle and late stages of the logarithmic growth period. At this stage, their metabolism was vigorous and the number of cells was sufficient. When cells at this stage were inoculated into SHM, the highest EC yield was obtained. The cells were in the stable growth phase and declining growth period at 30–42 and 42–54 h, respectively. At these stages, their metabolic activity was lower than that of cells at metaphase of logarithmic growth and the number of cells was insufficient, leading to decreased EC yields.

#### Effect of Sugar Content on EC Production

Glucose, maltose, and a small amount of dextrin are produced from sorghum materials after treatment with amylase and glucosidase. These carbon sources provide energy not only for growth, but also for metabolic processes that produce esters and other compounds. The Brix value is an index of the soluble sugars content of sorghum after enzymatic hydrolysis. The higher the Brix value, the higher the soluble sugars content in SHM. The yield of EC by YX3307 was low when the sugar content was low (data not shown), because the synthesis of intermediate metabolites and the expression of enzymes related to ester synthesis was limited, and there was limited growth and reproduction of yeast cells. As the sugar content increased, the cell biomass, concentration of metabolic intermediates, and expression of enzymes related to ester synthesis increased, leading to higher yields of EC. When the sugar content was 10 Brix, the highest yield of EC was 17.0 mg/L. As the sugar content increased beyond 10 Brix, the EC yield did not change significantly. Therefore, a sugar content of 10 Brix was used in subsequent experiments.

#### Effect of Time of Ethanol Addition on EC Production

**FIGURE 4:**
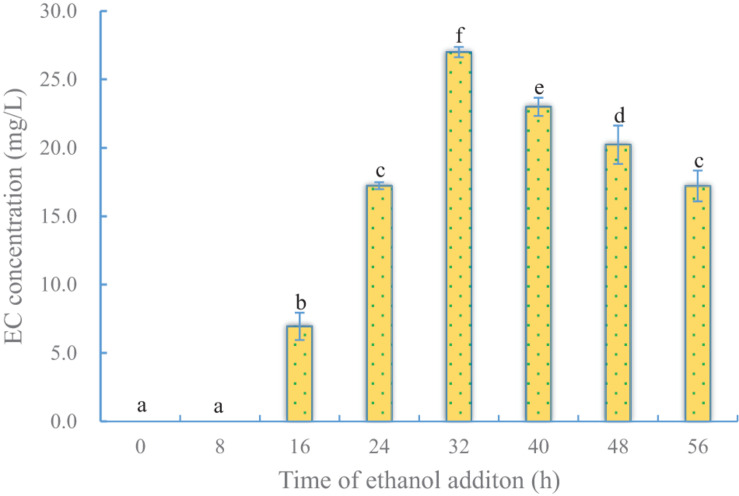
Effect of time of ethanol addition on EC production by YX3307. Same letters in the column indicates that the data do not differ significantly at 5% probability by the Tukey test.

To determine the effect of the timing of ethanol addition on EC production by YX3307, caproic acid was added at 24 h of culture, and ethanol was added at different time points as shown in Supplementary Table 3. Once any kind of precursor was added, the culture was kept in static and low-temperature conditions (20°C) to reduce the toxic effect of precursors on cells. After both the precursors were added, the fermentation was continued for 36 h. The results are shown in Figure 4. No EC was produced when ethanol was added before 16 h. This is not only because of the short fermentation period, but also because yeast cells were in the lag phase and logarithmic prophase (Supplementary Figure 5). During this period, EC could not be synthesized by YX3307 because of the toxicity of ethanol to cells (Figure 4). When yeast cells were in metaphase of logarithmic growth, the later phase of logarithmic growth, and the stable growth phase, they could tolerate a certain amount of ethanol. Therefore, they could convert the precursors (ethanol and caproic acid) into EC. The yield of EC was high when ethanol was added at metaphase and the later phase of logarithmic growth. A high yield of EC (27.0 mg/L) was obtained when ethanol was added at 32 h.

#### Effect of Inoculum Size on EC Production

Although there is little information available about the influence of inoculum size on ester synthesis, the results of a previous experimental study indicated that EC synthesis by YX3307 requires a sufficient biomass of cells in the optimal growth phase (Dufour et al., 2003). Therefore, the effect of inoculum size on the synthesis of EC by YX3307 was determined. There was no significant change in the yield of EC when the inoculum size was between 0.1 and 0.33% of the total volume (data not shown). As the inoculum size increased, the synthesis of EC also increased, and the highest production of EC (42.7 mg/L) was achieved with an inoculum size of 7.5%. With this inoculum size, the cell biomass was sufficient, the amount of harmful substances produced by YX3307 was small, and cells were metabolically active. In addition, the nutrient composition in the SHM was relatively rich. Cells can resist the toxic effect of precursors, such as caproic acid, and convert them into EC. However, as the inoculum size increases, the nutrients in SHM would be consumed too early because of the large biomass, and some harmful metabolites would be produced. This would reduce the ability of cells to resist precursors, leading to a decrease in the yield of EC. A larger inoculum would allow the yeast to resist the toxic effects of higher concentrations of precursors, which explains why the optimum inoculation size in this study was larger than that in most previous reports.

#### Effect of Timing of Caproic Acid Addition on EC Production

**FIGURE 5:**
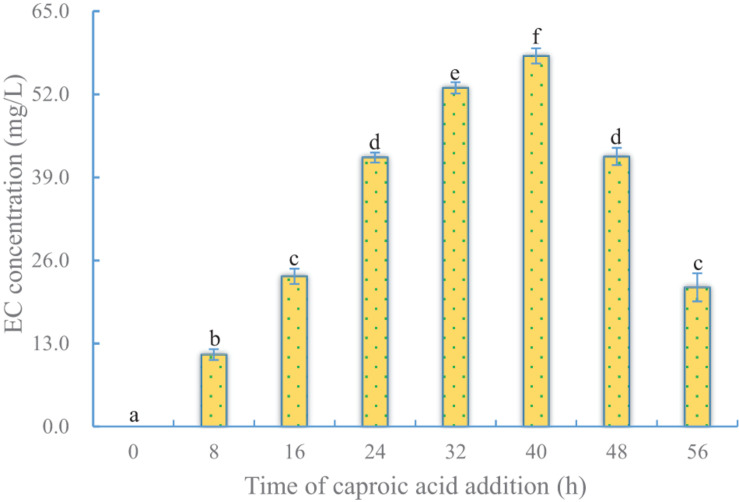
Effect of time of caproic acid addition on EC production by YX3307. Same letters in the column indicates that the data do not differ significantly at 5% probability by the Tukey test.

Just as the time point of ethanol addition was important for EC production, so was the time point of caproic acid addition. To test the effect of the timing of caproic acid addition on EC production, ethanol was added to the fermentation system at 32 h, while caproic acid was added at different times (Supplementary Table 3). Like in the case of ethanol addition, when caproic acid was added at the beginning, no EC was detected by 36 h after addition, indicating that the cells were sensitive to caproic acid at the early stage of fermentation. Higher yields of EC were obtained when caproic acid was added to cells at the middle and late logarithmic growth stage and the stable phase. The optimum time of caproic acid addition (40 h) was later than the optimum time for ethanol addition, because caproic acid was more toxic to cells than was ethanol (Figure 5 and Table 1).

#### Effect of Culture Time on EC Production

**FIGURE 6:**
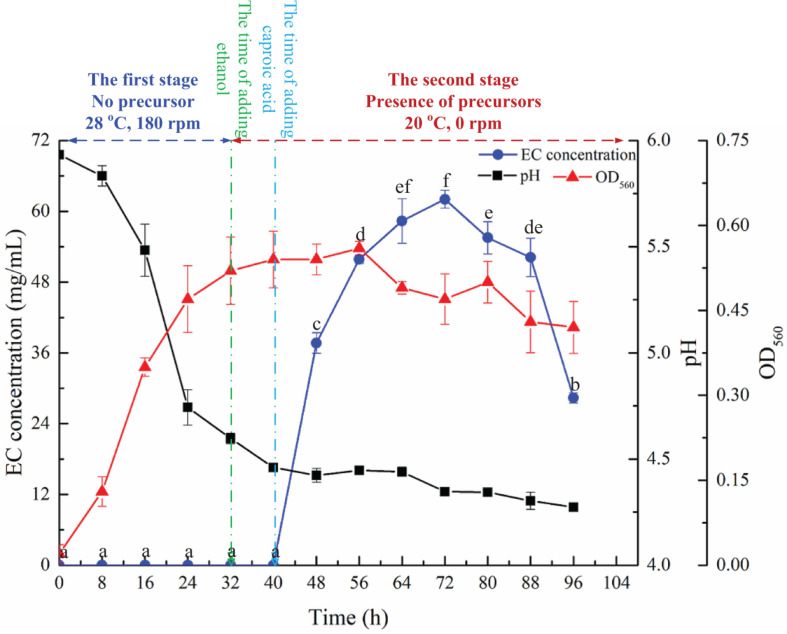
Effect of culture time on EC production by YX3307. Same letters in the column indicates that the data do not differ significantly at 5% probability by the Tukey test.

The time course of EC synthesis by YX3307 is shown in Figure 6. No EC was detected during 0–32 h, during which no precursors were added. That is, YX3307 could not synthesize EC without precursors. No EC was detected between 32–40 h when ethanol, but not caproic acid, was present, consistent with the results of previous studies (Figure 3). These results showed that the synthesis of EC by YX3307 differs from the synthesis of ethyl acetate by *W. anomalus* (Fan et al., 2018b). After adding caproic acid, EC production increased and then decreased over time. The production of EC was highest between 56 and 88 h, during which the highest yield was 62.0 mg/L at 72 h. Thus, compared with other reported EC-producing yeast strains, YX3307 has obvious advantages in terms of yield (Table 2).

**TABLE 2:** Summary of yeasts reported for EC yield.

**Strain**	**Origin**	**EC Yield (mg/L)**	**Year**	**References**
*Hanseniaspora uvarum* Yun268	Grapery	0.021	2018	Hu et al., 2018
*Saccharomyces cerevisiae*α5	−	0.5	2016	Chen et al., 2016
*Saccharomyces cerevisiae* 102SC	*Ziziphus mauritiana* fruits	110	2013	Nyanga et al., 2013
*Saccharomyces cerevisiae* 131SC		28.7		
*Saccharomyces cerevisiae* 153SC		92.0		
*Saccharomyces cerevisiae* 143SC		28.6		
*Saccharomyces cerevisiae* 135SC		21.3		
*Saccharomyces cerevisiae* 38SC		23.2		
*Pichia kudriavzevii* 125PK		1.4		
*Pichia kudriavzevii* 129PK		2.5		
*Pichia kudriavzevii* 166PK		2.5		
*Pichia fabianii* 65PF		1.3		
*Saccharomycopsis fibuligera* 66SF		1.8		
*Issatchenkia orientalis* W8Y-21	*Baijiu*-making workshop air	2.94 (g/L, after distilled of fermented grain)	2013	Wang et al., 2013
*Debaryomyces hansenii* G8M-28		3.10 (g/L, after distilled of fermented grain)		
*Debaryomyces hansenii* S532Y-20	Fermented grain	4.86 (g/L, after distilled of fermented grain)		
*Debaryomyces hansenii* S442Y-44		4.87 (g/L, after distilled of fermented grain)		
*Debaryomyces hansenii* Z8Y-13	*Baijiu*-making workshop air	7.50 (g/L, after distilled of fermented grain)		
*Zygosaccharomyces bailii* H1Y-24	Pit mud	2.53 (g/L, after distilled of fermented grain)		
*Issatchenkia orientalis* S433Y-17	Fermented grain	2.54 (g/L, after distilled of fermented grain)		
*Trichosporon coremiiforme* Z8Y-32	*Baijiu*-making workshop air	2.56 (g/L, after distilled of fermented grain)		
*Saccharomyces cerevisiae*	*Baijiu* distillery	0.21	2013	Wu et al., 2013
*Galactomyces geotrichum*		0.005		
*Kazachstania exigua*		0.13		
*Schizosaccharomyces pombe*		0.38		
*Saccharomyces cerevisiae* Kyokai no. 7	–	1.0	2004	Aritomi et al., 2004
*Hansenula mrakii* IFO 0895	–	0.03 mg/kg	1994	
*Clavispora lusitaniae* YX3307	*Daqu*	62.0	2020	This study

*“–,” no details.*

#### Preliminary Study on EC Synthesis

Four groups of tests were designed to preliminarily analyze the mechanism of EC synthesis by YX3307. The results are shown in Supplementary Figure 9. The pH value of SHM decreased when YX3307 consumed the soluble sugars. The lower pH conditions may have favored the formation of EC by the chemical reaction (esterification) between ethanol and caproic acid. However, no EC was detected in group A, indicating that EC was not produced by ethanol and caproic acid during fermentation with YX3307. The highest yield (61.7 mg/L) of EC was obtained in group B. By comparing the results from groups A and B, it can be concluded that EC is synthesized via a metabolic process, that is, the result of enzymatic catalysis, as was also confirmed in the previous optimization results. Compared with group B, group C produced little EC (0.1 mg/L), indicating that YX3307 produced almost no extracellular enzyme to convert ethanol and caproic acid to EC without precursors. In other words, the enzyme that catalyzes the synthesis of EC from ethanol and caproate acid by YX3307 was induced after addition of precursors, and/or was located inside the cell or on the cell membrane. In group D, some EC (3.8 mg/L) was detected in the presence of precursors after cells were added to new SHM. The higher yield in group D than in group C suggested that an extracellular enzyme for EC synthesis was induced by ethanol and caproic acid, or that the catalytic enzyme was located intracellularly or on the cell membrane. The EC yield was lower in group D than in group B, which may be due to the interference of cell treatments. These findings, combined with the results of the optimization of fermentation conditions, suggest that the enzyme that synthesizes EC is located intracellularly or on the cell membrane. A previous study demonstrated the importance of ethanol hexanoyl transferase in EC production (Lilly et al., 2006; Saerens et al., 2006; Chen et al., 2014). Precious studies have indicated that the alcohol acyltransferases EEB and EHT are the two most important enzymes for EC synthesis (Saerens et al., 2006; Takahashi et al., 2017). Although only two alcohol acyltransferases have been identified to synthesize EC so far, there may be other enzymes that synthesize EC in yeasts, and the key enzymes for EC synthesis may differ among different in different strains (Saerens et al., 2006). Thus, further studies are needed to clarify the intrinsic mechanism of EC synthesis by YX3307. The results of the present study provide an important experimental basis for studying EC biosynthesis.

#### Aroma Production

A total of 30 flavor compounds were detected by SPME-GC-MS in SHM after fermentation of YX3307 for 72 h. These flavor compounds included 12 alcohols, nine esters, two aldehydes, one phenol, one acid, one furan, three alkanes, and one ketone. Only 10 flavor compounds were detected in SHM, namely, one alcohol, four esters, one aldehyde, one furan, one ketone, and two other compounds (Supplementary Table 6). After comparing the flavor compounds between SHM and SHM fermented by YX3307, we concluded that: (i) the types and amounts differed between the two groups, with only four flavor compounds in both of them; (ii) six flavor compounds in SHM were not detected after culture with YX3307, indicating that they were metabolized and transformed into other flavor compounds; (iii) the 26 new flavor compounds in SHM were produced by activities of YX3307 using sugars or other nutrients as substrates; (iv) the contents of the four flavor compounds found in both groups were higher after fermentation with YX3307 because of its activity. Therefore, YX3307 can produce many volatile flavor compounds in SHM. Like other aroma-producing yeasts, YX3307 can also produce some higher alcohols, esters, aldehydes, and other flavor compounds, which can give alcoholic drinks unique characteristics (Bruner and Fox, 2020). Isobutanol, isoamyl alcohol, and furfuralcohol are important higher alcohols in *Baijiu*. When their content is less than 30 mg/L, they can impart sweetness and enhance the fragrance of other flavor compounds (Luo et al., 2015). They are also important precursors of other flavor compounds (Fan et al., 2019). There are few reports about linalool in *Baijiu*, but it is an important flavor compound in wine, conferring bergamot, lavender, and rose floral notes due to it relatively low odor perception thresholds (15 μg/L) (Li Z. H. et al., 2019; Lukic et al., 2020; Zhang X. Y. et al., 2020). Other yeasts and fungi also can produce linalool, which imparts a tea-flavor to *Baijiu* (Cui X. X. et al., 2019; Fan et al., 2019). 3-Methylthiopropanol, which has onion and meat odors, is an important contributor to the sesame aroma of *Baijiu* (Hong et al., 2020). Although YX3307 produced only small amounts of 3-methylthiopropanol, its olfactory threshold is very low. Thus, YX3307 can contribute to several characteristic flavors of *Baijiu*. (R)-(+)-β-Citronellol, geraniol bisabolol, and *trans*-nerolidol are important monoterpenols in wines and beer, and they are also present in *Baijiu* (Fan and Xu, 2013; Quan et al., 2013; Lukic et al., 2020). These terpenes can not only endow *Baijiu* with unique floral and fruit aromas, but also reduce the harm of ethanol (Sun et al., 2016; Li Z. H. et al., 2019). Some microorganisms can synthesize terpenes in the *Baijiu* fermentation environment, such as *S. cerevisiae* YF1914, *Eurotium chevalieri* CICC 41584, and YX3307 (as demonstrated in this study) (Cui X. X. et al., 2019; Fan et al., 2019). These microorganisms have potential applications in technologies to improve the health properties, flavor, and quality of *Baijiu* (Cui X. X. et al., 2019; Zhang Y. J. et al., 2020). Phenethyl alcohol with honey, spice, rose, and lilac flavors is an important flavor compound in many fermented products (Lin et al., 2020). The tastes of consumers change over time, and modern consumers tend to prefer slightly sweet, low-alcohol *Baijiu* (Han et al., 2013). YX3307 has potential applications in the development of sweet *Baijiu* because it has a high yield of phenethyl alcohol. From the aspect of ester production, YX3307 did not produce EC in the absence of caproic acid, but it produced only a small amount of ethyl acetate when acetic acid was not added. In addition, it produced phenylethyl acetate, which has a floral aroma, and 2-methyl butyric acid-2-ethyl phenyl ester, which has a tea aroma (Chen et al., 2018; Wei et al., 2020). YX3307 also produced benzaldehyde, 2, 4-dimethylbenzaldehyde, and 2, 3-dihydrobenzofuran in SHM. Previous studies have reported that benzaldehyde with almond and burnt-sugar flavors and a high odor intensity makes a major contribution to the caramel aroma of *Baijiu* (Yu et al., 2019). Similar to phenethyl alcohol, benzaldehyde is related to the sweet attributes of alcoholic drinks (Yu et al., 2019). The style of *Baijiu* is also affected by 2, 4-dimethylbenzaldehyde, which imparts an almond flavor, and 2, 3-dihydrobenzofuran, which imparts an oily incense flavor (Wu, 2008; Gao et al., 2014). In summary, the advantages of YX3307 are that it can synthesize EC and improve the flavor and quality of *Baijiu*.

## Conclusion

A yeast with high yield of EC was isolated from *Daqu*, and was identified as *C. lusitaniae*. The strain showed high tolerance to sodium chloride, glucose, and EC, and showed outstanding advantages in terms of EC synthesis. The highest yield of EC (62.0 mg/L) was obtained under the following conditions: culture of YX3307 for 32 h in SHM at 28°C with shaking at 180 rpm, followed by addition of 10% anhydrous ethanol, fermentation at 20°C in the static state, addition of 0.04% caproic acid at 40 h of culture, then fermentation until 72 h. The brix value of the medium was 10° and the initial pH was 6.0, the inoculum age was 30 h, and the inoculum size was 7.5%. YX3307 can also produce many other flavor compounds, such as β-phenethyl alcohol and terpenes, which are considered to be important for *Baijiu* quality. *C. lusitaniae* YX3307 has potential applications to improve the quality of *Baijiu*. For example, *Daqu* rich in *C. lusitaniae* YX3307 could be preparation for *Baijiu* production, and the fermentation process could be modified to favor the growth of *C. lusitaniae* YX3307 to obtain raw *Baijiu* with a high EC content.

## Data Availability Statement

The original contributions generated for this study are included in the article/Supplementary Material, further inquiries can be directed to the corresponding author.

## Author Contributions

GF and PL performed the experiments, analyzed the data, and wrote the manuscript. XC, HY, and YG helped to modify the graphs. LC and CT assisted the manuscript checking. XL provided assistance and guidance throughout the research. All authors contributed to the article and approved the submitted version.

## Conflict of Interest

XC was employed by the company Angel Yeast Co., Ltd. The remaining authors declare that the research was conducted in the absence of any commercial or financial relationships that could be construed as a potential conflict of interest.

## Footnotes

1 https://www.ncbi.nlm.nih.gov/

## Abbreviations

EC: ethyl caproate
GC-MS: gas chromatography-mass spectrometry
HS-SPME: headspace solid-phase microextraction
OD: optical density
SHM: sorghum hydrolysate medium
WL medium: Wallerstein laboratory medium
YPD medium: yeast extract peptone dextrose medium
NYGA medium: nutrient yeast glycerol agar medium.
