# Supplementary material for: Isolation and Identification of a High-Yield Ethyl Caproate-Producing Yeast From Daqu and Optimization of Its Fermentation
Source: Front Microbiol. 2021 May 31;12:663744. doi: 10.3389/fmicb.2021.663744 (PMC8200637; doi:10.3389/fmicb.2021.663744)
Supplement: Supplementary file 1 [file Data_Sheet_1.zip › Supplementary Figures.DOCX]

**Supplementary material for**

Isolation and identification of a high-yield ethyl caproate-producing yeast from *Daqu* and optimization of its fermentation

**FIGURE LEGENDS**

**SUPPLEMENTARY FIGURE S1** | The metabolic characteristics of YX3307 after cultured 3 days and the result of its identification by Biolog.

**SUPPLEMENTARY** **FIGURE S2** | Neighbor-joining phylogenetic tree based on 26S rDNA (D1/D2 region) gene sequence of strain YX3307 and its closest relative species.

**SUPPLEMENTARY** **FIGURE S3** | Effect of pH on EC production by YX3307. Same letters in the column indicates that the data do not differ significantly at 5% probability by the Tukey test.

**SUPPLEMENTARY FIGURE S4** | Effect of shaking speed on EC production by YX3307. Same letters in the column indicates that the data do not differ significantly at 5% probability by the Tukey test.

**SUPPLEMENTARY FIGURE S5** | The growth curve (circled line) of YX3307 and the change of pH (rectangle line) of SHM under static (blue line) and shaking conditions (red line).

**SUPPLEMENTARY FIGURE S6** | Effect of temperature on EC production by YX3307. Same letters in the column indicates that the data do not differ significantly at 5% probability by the Tukey test.

**SUPPLEMENTARY FIGURE S7** | Effect of inoculum age on EC production by YX3307. Same letters in the column indicates that the data do not differ significantly at 5% probability by the Tukey test.

**SUPPLEMENTARY FIGURE S8** | The growth curve of YX3307 cultured in 28 ^o^C incubator at 180 rpm in PDA.

**SUPPLEMENTARY FIGURE S9** | EC production in different groups by YX3307. Same letters in the column indicates that the data do not differ significantly at 5% probability by the Tukey test.


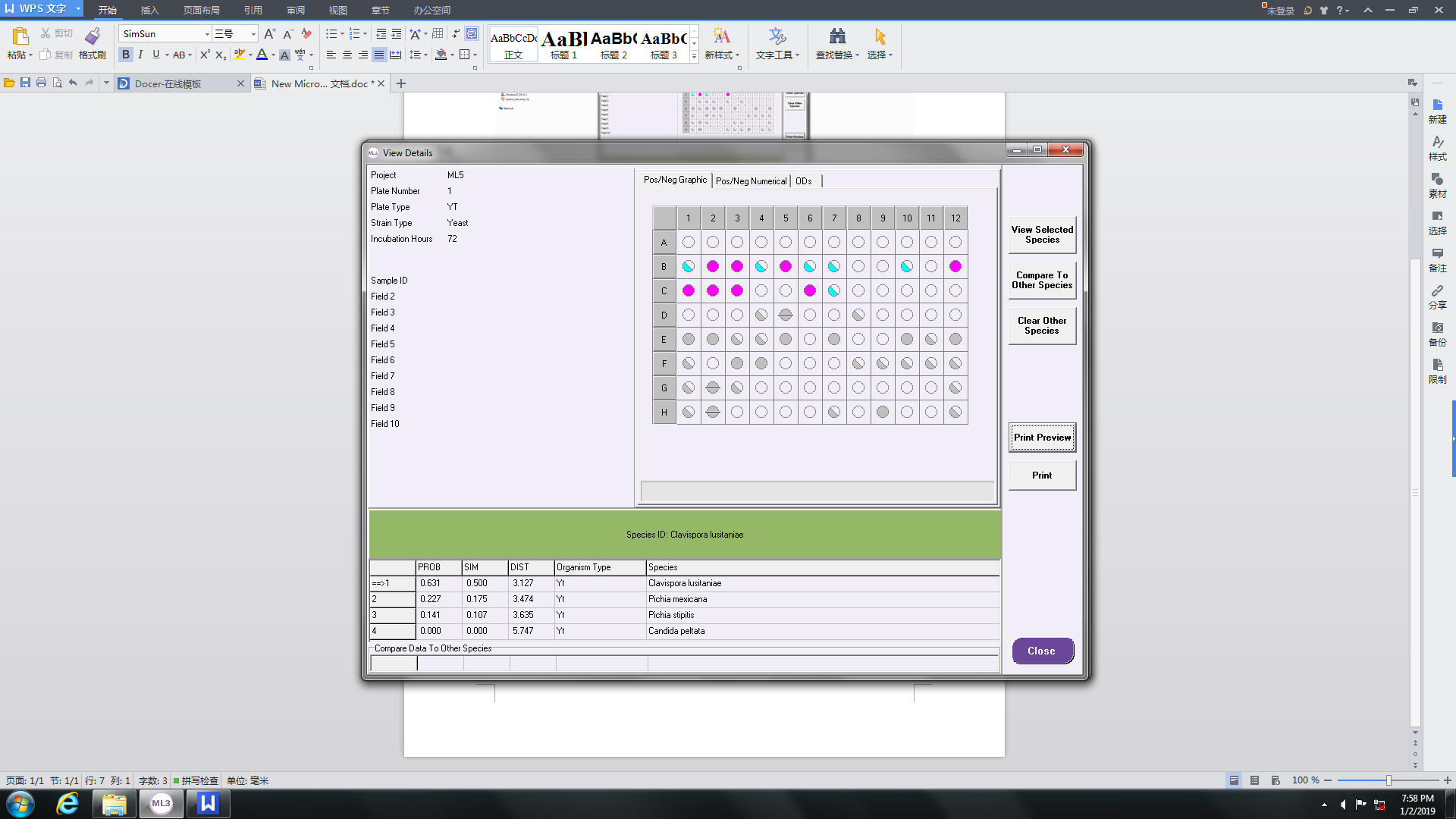


**SUPPLEMENTARY FIGURE S1** | The metabolic characteristics of YX3307 after cultured 3 days and the result of its identification by Biolog.

AY894824.1 *Clavispora lusitaniae* EXOC34

KC616317.1 *Clavispora lusitaniae* APKU-3

EF063126.1 *Clavispora lusitaniae* EXOC7

FJ627991.1 *Clavispora lusitaniae* FSMP-Y37

EU669469.1 *Clavispora lusitaniae* 7MCHS

EF063129.1 *Clavispora lusitaniae* ACE6

AM397859.1 *Clavispora* sp. YS 109

GQ396267.1 *Clavispora lusitaniae* ExoC21

AJ508571.1 *Clavispora lusitaniae* CBS 4413T

DQ377645.1 *Clavispora lusitaniae* VTT C-04533

GU904175.1 *Clavispora* sp. BM6-1

**YX3307**

KF830171.1 *Clavispora lusitaniae* MB141

EF536910.1 *Clavispora lusitaniae* GSWW10

97

46

47

25

19

49

94

68

94

0.5

**SUPPLEMENTARY** **FIGURE S2** | Neighbor-joining phylogenetic tree based on 26S rDNA (D1/D2 region) gene sequence of strain YX3307 and its closest relative species.

b


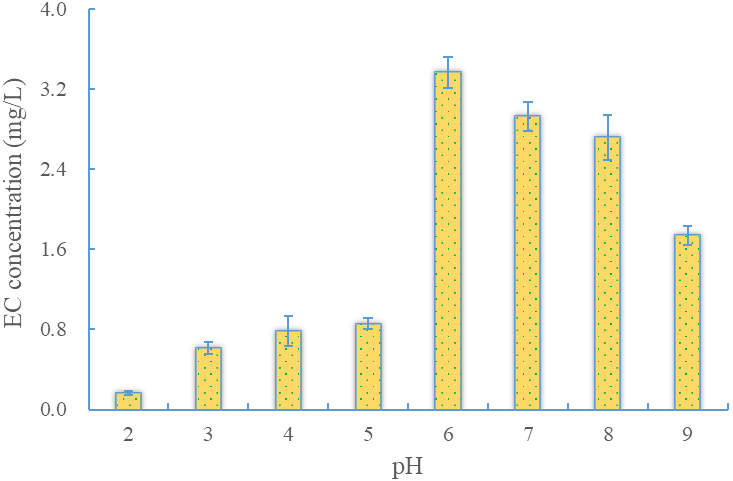


a

c

c

f

e

e

d

**SUPPLEMENTARY FIGURE S3** | Effect of pH on EC production by YX3307. Same letters in the column indicates that the data do not differ significantly at 5% probability by the Tukey test.


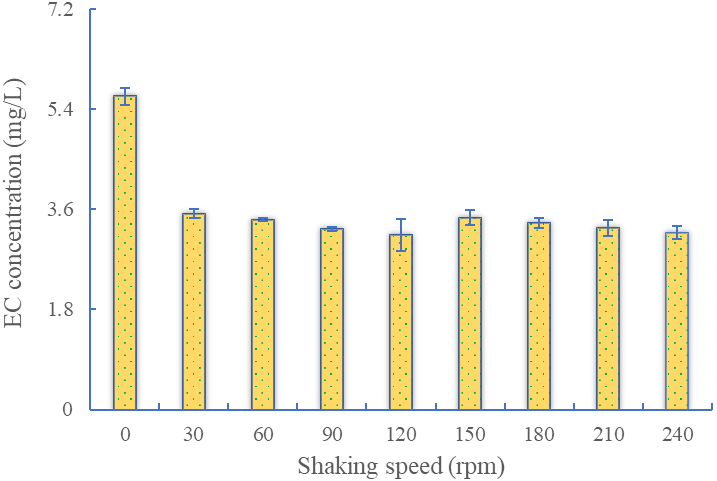


b

a

a

a

a

a

a

a

a

**SUPPLEMENTARY FIGURE S4**| Effect of shaking speed on EC production by YX3307. Same letters in the column indicates that the data do not differ significantly at 5% probability by the Tukey test.





**SUPPLEMENTARY FIGURE S5** | The growth curve (circled line) of YX3307 and the change of pH (rectangle line) of SHM under static (blue line) and shaking conditions (red line).


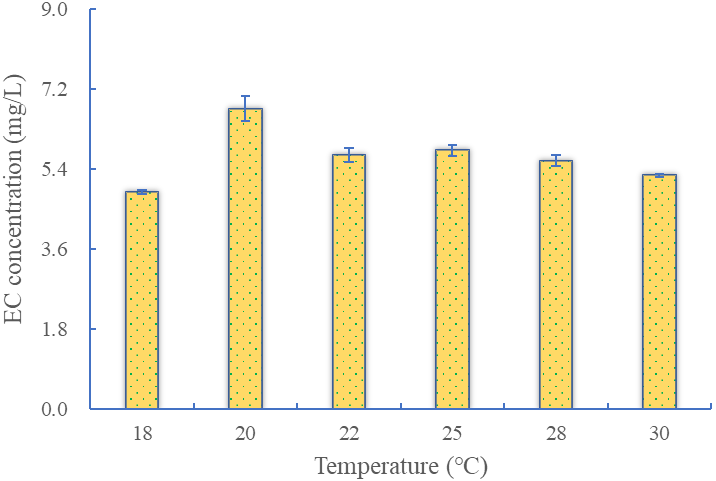


a

d

c

c

c

b

**SUPPLEMENTARY FIGURE S6** | Effect of temperature on EC production by YX3307. Same letters in the column indicates that the data do not differ significantly at 5% probability by the Tukey test.


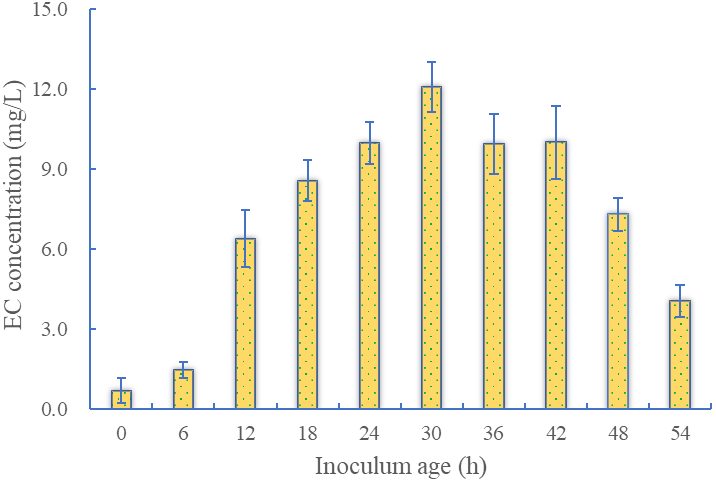


a

a

c

cd

d

e

d

de

c

b

**SUPPLEMENTARY FIGURE S7** | Effect of inoculum age on EC production by YX3307. Same letters in the column indicates that the data do not differ significantly at 5% probability by the Tukey test.

**SUPPLEMENTARY FIGURE S8** | The growth curve of YX3307 cultured in 28 ^o^C incubator at 180 rpm in PDA.


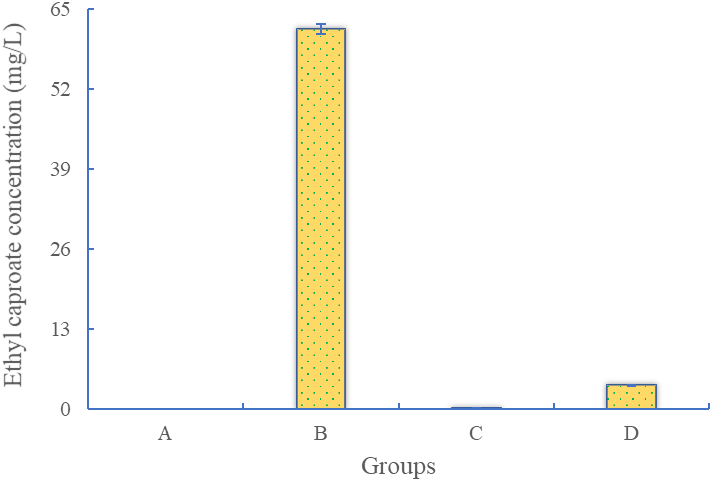


a

d

b

c

**SUPPLEMENTARY FIGURE S9** | EC production in different groups by YX3307. Same letters in the column indicates that the data do not differ significantly at 5% probability by the Tukey test.
