# Supplementary material for: Isolation and Identification of a High-Yield Ethyl Caproate-Producing Yeast From Daqu and Optimization of Its Fermentation
Source: Front Microbiol. 2021 May 31;12:663744. doi: 10.3389/fmicb.2021.663744 (PMC8200637; doi:10.3389/fmicb.2021.663744)
Supplement: Supplementary file 1 [file Data_Sheet_1.zip › Supplementary Tables.DOCX]

**Supplementary material for**

Isolation and identification of a high-yield ethyl caproate-producing yeast from *Daqu* and optimization of its fermentation

**SUPPLEMENTARY TABLE S1** | Detailed information of *Daqu* samples

| **Sample code** | **Type** | **Raw materials** | **Collection time** | **Source** |
| --- | --- | --- | --- | --- |
| D1 | Medium temperature | Wheat | Apr, 2016 | Wuliangye Group Co. Ltd. |
| D2 | Medium temperature | Wheat, corn, sorghum, rice and sticky rice | Apr, 2016 | Gujing Gongjiu Co. Ltd. |
| D3 | High temperature |  | Sep, 2016 |  |
| D4 | High temperature | Wheat | May, 2017 | Yanghe Distillery Co. Ltd. |
| D5 | Medium temperature | Wheat | Jul, 2017 | Gucheng Laojiao Co. Ltd. |
| D6 | Medium temperature | Wheat | Oct, 2017 | Bancheng Shaoguo Co. Ltd. |
| D7 | High temperature | Wheat | Oct, 2017 | Luzhou Laojiao Co. Ltd. |
| D8 | Medium temperature |  |  |  |
| D9 | Medium temperature | Wheat and barley | Oct, 2017 | Jiannanchun Co. Ltd. |
| D10 | Medium temperature | Wheat | Apr, 2018 | Daohuaxiang Co. Ltd. |
| D11 | Medium temperature | Wheat | Apr, 2018 | Yimeng Laojiu Co. Ltd. |
| D12 | Medium temperature | Wheat | May, 2018 | Bandaojing Distillery Co. Ltd. |
| D13 | Medium temperature | Wheat, barley and pea | Jun, 2018 | Jinzhongzi Distillery Co. Ltd. |
| D14 | Medium temperature | Wheat | Sep, 2018 | Songhe Distillery Co. Ltd. |
| D15 | High temperature |  |  |  |

**SUPPLEMENTARY TABLE S2** | Factors and levels for growth or tolerance test design

| **Factor** | **Level** |
| --- | --- |
| Temperature (℃) range for growth | 20, 25, 30, 35, 40, 45 and 50 |
| pH range for growth | 1, 2, 3, 4, 5, 6, 7, 8, 9, 10,11 and 12 |
| Glucose tolerance test (%, v/v) | 10, 20, 30, 40, 50, 60, 70, 80 and 90 |
| NaCl tolerance test (%, w/v) | 0, 5, 10, 15, 20, 25 and 30 |
| Ethanol tolerance test (%, v/v) | 0, 2, 4, 6, 8, 10, 12, 14, 16, 18 and 20 |
| Caproic acid tolerance test (%, v/v) | 0, 0.02, 0.04, 0.06, 0.08, 0.10 and 0.12 |
| Ethyl caproate tolerance test (mg/L) | 0, 300, 600, 900, 1200, 1500 and 1800 |

**SUPPLEMENTARY TABLE S3** | Factors and levels of single factor design for EC production

| **Factor** | **Level** |
| --- | --- |
| pH | 4, 5, 6, 7, 8 and 9 |
| Shaking speed (rpm) | 0, 30, 60, 90 and 120 |
| Temperature (℃) | 18, 20, 22, 25, 28 and 30 |
| Ethanol concentration (%) | 0, 2, 4, 6, 8, 10, 12, 14 and 16 |
| Caproic acid concentration (%) | 0, 0.02, 0.04, 0.06, 0.08 and 0.1 |
| Inoculum age (h) | 0, 6, 12, 18, 24, 30, 36, 42, 48 and 54 |
| Sugar content (Brix) | 2, 4, 6, 8, 10, 12 and 14 |
| Time of ethanol addition (h) | 0, 8, 16, 24, 32, 40, 48 and 56 |
| Inoculum size (%, v/v) | 0.1, 0.3, 0.5, 1.0, 2.0, 5.0, 7.5 and 10.0 |
| Time of caproic acid addition (h) | 0, 8, 16, 24, 32, 40, 48 and 56 |
| Culture time (h) | 0, 8, 16, 24, 32, 40, 48, 56, 64, 72, 80, 88 and 96 |

**SUPPLEMENTARY TABLE S4** | The yeasts for EC production exceeding 0.5 g/L in our study

| No. | EC production (g/L) | No. | EC production (g/L) |
| --- | --- | --- | --- |
| YX3307 | **3.4±0.3** | Y8#29 | 0.7±0.0 |
| YF1914 | 1.3±0.2 | F1607 | 0.8±0.0 |
| F12501 | 1.1±0.1 | X3401 | 0.6±0.0 |
| Y7#16 | 1.1±0.2 | Y1801 | 0.8±0.1 |
| F12504 | 1.2±0.1 | F1612 | 0.6±0.0 |
| Y4#14 | 0.6±0.0 | F10511 | 0.7±0.1 |
| Y8#01 | 0.5±0.0 | F3303 | 0.8±0.0 |
| Y51 | 0.5±0.1 | F4401 | 1.2±0.2 |
| F3301 | 0.5±0.1 | F13026 | 0. 9±0.1 |
| Y4#16 | 0.5±0.0 |  |  |

**SUPPLEMENTARY TABLE S5** | The ability of YX3307 to synthesize ethyl acetate, ethyl lactate, ethyl butyrate, ethyl caproate and ethyl octanoate

| Esters | Precursors | Yield (mg/L) |
| --- | --- | --- |
| Ethyl ester | 4% (v/v) ethanol and 0.02% (v/v) acetic acid | 0.25 |
| Ethyl lactate | 4% (v/v) ethanol and 0.02% (v/v) lactic acid | - |
| Ethyl butyrate | 4% (v/v) ethanol and 0.02% (v/v) butyric acid | - |
| Ethyl caproate | 4% (v/v) ethanol and 0.02% (v/v) caproic acid | 3.37 |
| Ethyl octanoate | 4% (v/v) ethanol and 0.02% (v/v) octanoic acid | - |

Note: “-”: not detected.

**SUPPLEMENTARY TABLE S6** | The volatile compounds in SHM with or without YX3307 (µg/L)

| Volatile compounds | SHM | YX3307 |
| --- | --- | --- |
| Ethanol | - | 350±31 |
| Isobutanol |  | 61±7 |
| Linalool |  | 41±3 |
| 3-Methylthiopropanol |  | 22±5 |
| (R)-(+)-β-Citronellol |  | 13±4 |
| Geraniol |  | 11±3 |
| Bisabolol |  | 21±4 |
| Trans-nerolidol |  | 12±3 |
| Isoamyl alcohol | - | 554±27 |
| β*-*Phenethyl alcohol | 7±1 | 1092±43 |
| Furfuralcohol | - | 13±3 |
| (R)-3, 7-Dimethyl-6-octenol | - | 22±6 |
| Σ Alcohols | **7** | **2,212** |
| Ethyl acetate | - | 143±13 |
| Phenylethyl acetate | - | 131±26 |
| 2-Methyl butyric acid-2-ethyl phenyl ester | - | 41±6 |
| α-Amyl-γ-butyrolactone | ^-^ | 11±2 |
| Ethyl retrooleate | 57±4 | - |
| γ-Decalactone | ^-^ | 42±3 |
| Ethyl linolenate | 84±13 |  |
| Dibutyl phthalate | 2±1 | 13±3 |
| Isopropyl palmitate | - | 22±4 |
| γ-nonalactone | ^-^ | 14±2 |
| Ethyl palmitate | 116±21 |  |
| 2,2,4-Trimethyl-1,3-pentanediol diisobutyrate | ^-^ | 54±7 |
| Σ Esters | **259** | **471** |
| Benzaldehyde | ^-^ | 24±4 |
| 2, 4-Dimethylbenzaldehyde | 2±1 | 43±12 |
| Σ Aldehydes | **2** | **67** |
| 2,4-Ditertiary butyl phenol | ^-^ | 44±9 |
| Σ Phenols | **-** | **44** |
| Isobutyric acid | ^-^ | 31±7 |
| Σ Acids | **-** | **31** |
| 2, 3-Dihydrobenzofuran | 7±3 | 34±6 |
| Σ Furans | **-** | **34** |
| Tetradecane | ^-^ | 1±0 |
| Hexadecane | ^-^ | 11±3 |
| 2,6,10, 14-Tetramethylpentadecane | ^-^ | 1±0 |
| Σ Alkanes | **-** | **12** |
| 2-Methyl-4-heptanone | 20±4 |  |
| 3-Hydroxy-2-butanone | - | 13±2 |
| Σ Ketones | **20** | **13** |
| Benzothiazole | 54±7 | - |
| (2-Aziridinylethyl) amine | 2±1 | - |
| Σ Others | **56** | **-** |
| Sum | **364** | **2,884** |

SHM: the volatile compounds in SHM without YX3307; YX3307: the volatile compounds in SHM with YX3307.

Note: Data are average of three replicates ± standard deviations; “-”, not detected.
